# Two Cases of Cartilaginous Choristoma—Not Chondroma of the Bony External Auditory Canal

**DOI:** 10.1155/2018/6346453

**Published:** 2018-12-11

**Authors:** Kohei Yamahara, Yuki Katsura, Yuki Egawa, Kana Lee, Satoshi Ikegami

**Affiliations:** ^1^Department of Otolaryngology, Head and Neck Surgery, Shizuoka City Shizuoka Hospital, Shizuoka City, Shizuoka 420-8630, Japan; ^2^Department of Pathology, Shizuoka City Shizuoka Hospital, Shizuoka City, Shizuoka 420-8630, Japan; ^3^Department of Otolaryngology, Shin-Suma General Hospital, Kobe, Hyogo 654-0048, Japan

## Abstract

The presence of a cartilaginous mass on the bony external auditory canal is an unusual finding. Currently, two different diagnoses have been used to describe this type of mass: chondroma and cartilaginous choristoma. There is currently no consensus on which diagnosis is appropriate for this type of lesion. Choristoma is defined as a tumor-like growth of normal tissue occurring in an abnormal location. Histological examination alone cannot be used to distinguish between cartilaginous choristoma and chondroma, as both lesions comprise normal mature hyaline cartilage. To diagnose a mass as cartilaginous choristoma on the bony external auditory canal, it is necessary to confirm that it does not originate from the underlying periosteum. Here, we present the cases of two patients with typical cartilaginous masses on the bony external auditory canal, in which the surgical findings showed that the masses were not in contact with the underlying periosteum, indicating that cartilaginous choristoma—not chondroma—is an appropriate diagnosis for these mass lesions. The clinical findings (characteristic appearance and location) reported here may aid clinicians in the diagnostic and surgical management of these cartilaginous masses.

## 1. Introduction

The presence of a cartilaginous mass on the bony external auditory canal (EAC) is an unusual finding, with as few as 53 cases reported in the literature [1–7]. Previously, authors referred to these masses as *chondromas* in their reports [1, 2, 4, 7]. However, in 2005, Lee proposed that these masses should be diagnosed as *cartilaginous choristomas* (CC) rather than chondromas [3]. Nevertheless, nearly all reports continue to refer to these lesions as chondromas [5, 6]; thus, there is currently no consensus regarding how to appropriately identify these masses. Choristoma is defined as a benign tumor-like growth of normal tissue occurring in an abnormal location [8, 9]. There have been numerous reports of various types of choristomas occurring within the head and neck. They include gastric mucosa in the tongue [10], salivary gland tissue in the middle ear [11], and osseous or cartilaginous masses in the intraoral soft tissues [9]. Histopathological examination alone cannot distinguish between CC and chondroma because both mass lesions are composed of normal mature hyaline cartilage. To diagnose a tissue mass as CC on the bony EAC, it is necessary to confirm that it does not originate from the bony EAC. In this report, we present the cases of two patients with typical cartilaginous masses on the bony EAC, in which the surgical findings showed that the masses were not in contact with the underlying periosteum of the bony EAC, indicating that CC is an appropriate diagnosis for these types of lesions.

## 2. Case Presentations

**Figure 1:**
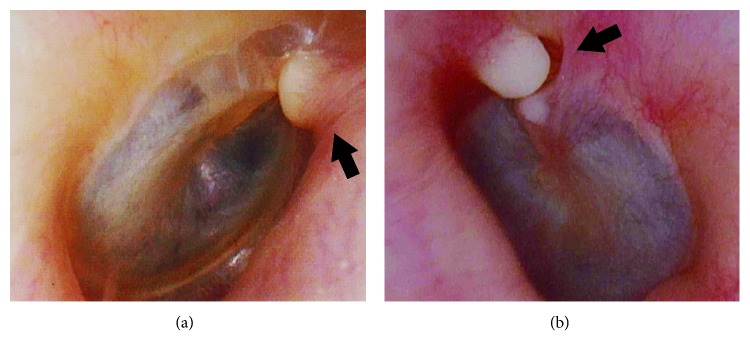
Otoscopic view of the right ear in Case 1 (a), and the left ear in Case 2 (b). Masses were discovered at the medial portion of the anterior wall of the bony EAC just in front of the short process of the malleus. Arrows indicate the masses.

**Figure 2:**
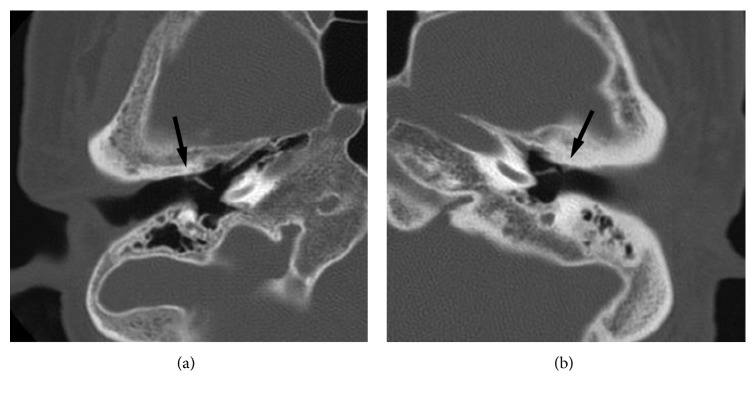
CT images of the temporal bone in Case 1 (a) and Case 2 (b). CT imaging shows a lesion emanating from the anterior bony EAC in both cases. In each case, the EAC appears flat with well-defined boundaries. Arrows indicate the masses.

**Figure 3:**
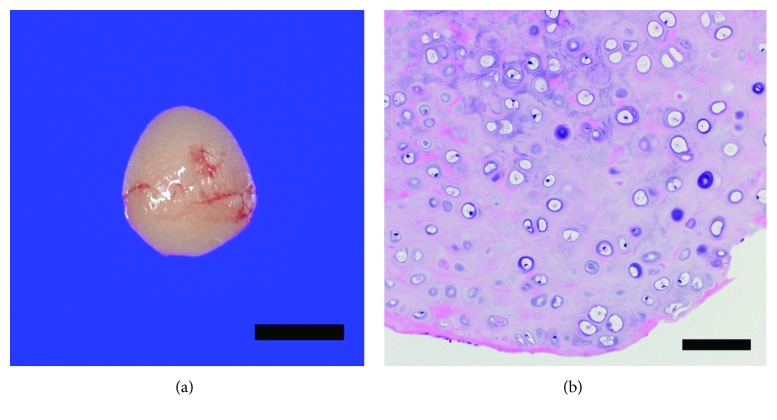
Gross and pathologic examination of the excised mass of Case 2. (a) The masses appear round with a smooth surface (bar represents 1 mm). (b) Pathologic examination shows the excised masses comprising mature hyaline cartilage formed by normal chondrocytes (bar represents 100 *μ*m).

Here, we report the cases of one 73-year-old woman (Case 1) and one 55-year-old man (Case 2). Both patients did not have a history of swimming in cold water or taking saunas. They each presented with a mass, discovered incidentally on routine examination, at the medial portion of the anterior wall of the bony EAC just in front of the short process of the malleus (Figures 1(a) and 1(b)). The mass was located in the right ear in Case 1 and in the left ear in Case 2; they did not touch the tympanic membranes, which were found to be normal in both patients. These masses were about 2 mm in diameter and were noted to be firm and white, somewhat mobile, and covered with normal appearing epithelium. Palpation using a Rosen needle revealed that these lesions were firm and nontender, suggesting that they were osseous or cartilaginous masses instead of soft tumors (i.e., cholesteatoma, keratoma, etc.). A CT scan of the temporal bone revealed a lesion emanating from the anterior bony EAC in both cases (Figures 2(a) and 2(b)). Additionally, CT imaging showed that the bony EAC was flat and had well-defined boundaries, suggesting that it had not been invaded. Although both patients were asymptomatic and the masses did not show any enlargement in size during the follow-up period (ranging from 3 to 4 years), both patients wanted the masses removed, and they were excised via the transcanal approach using a Rosen needle. The masses were located between the squamous epithelium and periosteum of the bony EAC, and they separated very easily—again indicating that masses were not in contact with the periosteum. On gross examination, the excised masses appeared round with a smooth surface (Figure 3(a)). After the masses were removed, the periosteum of the bony EAC just beneath the surgical site was found to be normal in both cases. The operation was completed without curettement of the underlying periosteum or bone cortex. Pathologic examination demonstrated that the excised masses consisted of mature hyaline cartilage formed by normal chondrocytes (Figure 3(b)). Based on information from the intraoperative report, the pathologist was informed that the masses did not arise from the periosteum, though they were on the surface of the bony EAC. Thus, based on these histological and intraoperative findings, we obtained the diagnosis of CC on the bony EAC—not chondroma. The postoperative courses of both patients were uneventful, and no recurrences were found during follow-up at 1 to 2 years postoperatively. Both patients described here provided written informed consent for the publication of this case report.

## 3. Discussion

Only 53 cases of a cartilaginous mass on the bony EAC have been documented in the literature [1–7]. However, with heightened awareness, an increasing number of such lesions were detected in our hospital, suggesting that this lesion is not as rare as previously thought. All but two of these reports have described patients of Asian ethnicity. Additionally, there appears to be an equal distribution between male and female patients, with an average age of 31.5 years at the time of diagnosis [5]. These lesions typically present as a single, white, hard, and round mass with a smooth surface and a diameter of 2 mm; moreover, they frequently occur in the medial portion of the anterior wall of the bony EAC, just in front of the short process and handle of the malleus. Although these masses may meet the criteria for CC, they are still frequently described as chondromas in the literature [5, 6]. In this report, we assessed the clinical and histological features of two consecutive cases of such lesions and confirmed the diagnoses these masses as CC—as suggested by Lee.

Chondromas can be classified into several types, depending on the sites in which they originate. When a cartilaginous mass is found at the surface of the bony EAC, just as in our cases, “periosteal” chondroma is considered in the differential diagnosis of CC [3]. Periosteal chondroma is a benign hyaline cartilage neoplasm of the bone surface that arises from the periosteum [12] and has the potential to grow [13]. Initially, this lesion is small and is located on the surface of the bone. As it enlarges, it erodes the underlying cortex, and in some cases, the periosteal chondroma can reach a diameter of 6 cm [13]. Because both the periosteal chondroma and CC consist of mature hyaline cartilage formed by normal chondrocytes, they cannot be distinguished based on pathological examination alone. Thus, intraoperative findings are essential for distinguishing between the two diagnoses; the underlying periosteum and cortex should be eroded in cases of periosteal chondroma [14], whereas the periosteum and cortex should be normal in cases of CC [3]. In the cases reported here, the masses did not appear to be in contact with the underlying periosteum, and there was no evidence of erosion in the periosteum of the bony EAC just beneath the masses, indicating that they did not originate from the periosteum. These findings led us to conclude that these masses were CC and not periosteal chondroma, a diagnosis which has been frequently reported in studies of cartilaginous masses of the bony EAC [1, 2, 4–7]. However, these reports also described that these masses were removed very easily with a pick, needle, or curette, just as in our cases, further suggesting that they may have been CCs rather than periosteum chondromas. On the other hand, there is only one report describing the case of a chondroma that involved the bone cortex of the bony EAC, and it was accompanied by facial nerve paralysis [7]. Considering the bony involvement of the mass, we can assume that the diagnosis of chondroma for that case was appropriate. Thus, chondromas arising from EAC may be far rarer than previously thought. The ability to distinguish between CC and periosteum chondroma, preoperatively or intraoperatively, is important. It is not only a matter of terminology, but the diagnosis also determines the extent of mass excision; in cases of CC, successful treatment is obtained by simple excision of the lesion, whereas in cases of periosteum chondroma, complete excision of lesion with curettement of the underlying bone is required to avoid recurrence. Because CC is not a well-known lesion and there are no histological differences between CC and chondroma, pathologists frequently diagnose the cartilaginous masses on the bony EAC as simple chondromas if they are not provided with any additional clinical or intraoperative information. Therefore, to obtain a more accurate pathological diagnosis, surgeons should provide information to the pathologist concerning whether the masses were in contact with the periosteum or whether the underlying periosteum was eroded.

The pathogenesis of CC is thought to be due to the migration of embryological cartilage cells [3]. It is speculated that the mass develops from heterotopic cartilaginous embryonic rests of Meckel's or Reichert's cartilage of the first or second branchial arch. The cartilage precursor cells mistakenly migrate into the primitive external ear canal during invagination of the first pharyngeal cleft and grow deep in the ear canal. However, their true origin remains unclear.

When the lesion occurs at the typical site (i.e., the medial portion of the anterior wall of the bony EAC), the differential diagnosis can be narrowed by first categorizing the lesion into two groups: soft mass (cholesteatoma, keratoma, etc.) or firm mass (CC, chondroma, chondrosarcoma, etc.). This step could be easily achieved by palpation of the mass using a tool, such as a Rosen needle. If the lesion is found to be a firm mass, a CT scan could be useful in distinguishing between CC, periosteum chondroma, and a malignant tumor; the characteristic radiographic feature of periosteum chondroma is erosion of the underlying cortex [14]. In both cases described in this report, a CT scan showed no apparent erosion of the cortex, suggesting the possibility of CC. However, sometimes CT imaging cannot clearly detect these masses, as they are usually very small. Additionally, if the mass is in the early stages of periosteum chondroma formation, the cortex may not yet be eroded, making it nearly impossible for a CT scan to distinguish between CC and periosteal chondroma based on this characteristic.

In the literature, the majority of reported CCs on the bony EAC were removed surgically; however, several authors have described that CCs have low growth potential and have not been reported to undergo malignant transformation. In consideration of this, observation is an appropriate strategy. Nonetheless, one study reported that a single CC enlarged in size and touched the tympanic membrane, resulting in conductive hearing loss [4]. Furthermore, it is very difficult to distinguish between CC and periosteal chondroma, preoperatively. Thus, watchful observation is needed, even in asymptomatic patients.

## 4. Conclusion

In this case study, the cartilaginous masses on the bony EAC were appropriately diagnosed as CC and not chondromas. Preoperative examination, which included palpation and CT imaging, and intraoperative findings were useful in obtaining a proper pathological diagnosis for these lesions.
